# The expression of endogenous voltage‐gated potassium channels in HEK293 cells is affected by culture conditions

**DOI:** 10.14814/phy2.13663

**Published:** 2018-04-17

**Authors:** Arturo Ponce, Aida Castillo, Lorena Hinojosa, Jacqueline Martinez‐Rendon, Marcelino Cereijido

**Affiliations:** ^1^ Department of Physiology, Biophysics and Neurosciences Center For Research and Advanced Studies (Cinvestav) México D. F. México

**Keywords:** 4AP, HEK293, potassium channels, TEA, trypsin

## Abstract

HEK293 cells are widely used as a host for expression of heterologous proteins; yet, little care has been taken to characterize their endogenous membrane components, including ion channels. In this work, we aimed to describe the biophysical and pharmacological properties of endogenous, voltage‐dependent potassium currents (IKv). We also examined how its expression depends on culture conditions. We used the electrophysiological technique of whole‐cell patch clamp to record ion currents from HEK293 cells. We found that HEK cells express endogenous, voltage‐dependent potassium currents. We also found that diverse culture conditions, such as the passage number, the cell density, the type of serum that complements the culture media and the substratum, affect the magnitude and shape of IKv, resulting from the relative contribution of fast, slow, and noninactivating component currents. Incubation of cells in mature monolayers with trypsin–EDTA, notoriously reduces the magnitude and modifies the shape of voltage‐dependent potassium endogenous currents; nonetheless HEK cells recover IKv′s magnitude and shape within 6 h after replating, with a process that requires synthesis of new mRNA and protein subunits, as evidenced by the fact that actinomycin D and cycloheximide, inhibitors of synthesis of mRNA and protein, respectively, impair the recovery of IKv after trypsinization. In addition to be useful as a model expression system, HEK293 may be useful to understand how cells regulate the density of ion channels on the membrane.

## Introduction

HEK‐293 is an immortalized cell line, of epithelial morphology, obtained by exposure of human embryonic kidney cells to sheared fragments of human adenovirus type 5 DNA (Graham et al. 1977). Given that these cells are very easy to grow and transfect, they have been used as a host model for expression and analysis of diverse heterologous proteins, such as receptors (Keith et al. 1996; Puett et al. 2005), pumps (Babichev et al. 2005; Chaâbane et al. 2006), and carriers (Dayem et al. 2008; Weinglass et al. 2008); They have been used also for production of synthetic polymer nanoparticles (Kim et al. 2005). Because this permanently transformed cell line has incorporated Ad5 into chromosome 19, it has been used for generation of recombinant E1‐deleted human adenoviral vectors (Thomas and Smart 2005) as well as retroviral‐based vectors (Silva et al. 2014).

HEK293 cells are also a preferred choice among electrophysiologists, who seek to study the biophysical properties of heterologous ion channels, due to its small size and voltage can be conveniently clamped. In addition, gigaseals are easily accomplished because HEK cells have no conspicuous processes. Therefore, HEK cells have been used to study the properties of a variety of exogenous ion channels, including sodium channels (Beyder et al. 2010; Mashimo et al. 2010; Lin et al. 2016), potassium channels (Aréchiga et al. 2008; Kimm and Bean 2014; Liu et al. 2014; Tang et al. 2015; Jiménez‐Pérez et al.^2016^), calcium channels (Pérez‐García et al. 1995; Perez‐Reyes et al. 1999; Bedford et al. 2016), chloride channels (Fuller et al. 2001; Sala‐Rabanal et al. 2015; Liantonio et al. 2016), transient receptor potential channels (Grimm et al. 2003; Sawada et al. 2007; Fernández et al. 2011), and aquaporins (Heo et al. 2008; Nishihara et al. 2011).

Despite being so widely used, relatively little attention has been taken to study the variety of endogenous ion channels that these cells express, let alone to understand the factors that determine and regulate its expression. Endogenous channels that had been described so far include voltage‐gated sodium and calcium channels (Berjukow et al.^1996^; He and Soderlund 2010), as well as TRP channels (Amarouch et al. 2013). Endogenous voltage‐dependent potassium currents have been described in HEK293, although with discrepant results: Zhu et al. (1998) reported that potassium channels have a minor contribution to the observed outward currents. Yu and Kerchner (1998) observed only delayed rectifier but no IA currents. Avila et al. (2004) reported at least two different types of voltage‐gated potassium channels, although they did not address their identity. Later on, Varghese et al. (2006) and Kurejová et al. (2007) reported that both IK‐ and IA‐type currents are endogenously expressed in these cells. In this work, we studied the functional and pharmacological properties of endogenous, voltage‐dependent potassium currents of HEK293 cells and how culture conditions influence their expression. We also studied how trypsinization of cells during the process of harvesting and subculturing affect these currents.

## Materials and Methods

### Culture of cells

HEK293 cells were cultured in disposable Petri dishes with a medium composed of Dulbecco's modified Eagle's (DMEM, GIBCO, 12800‐017), 10% fetal bovine serum (Gibco™ Fetal Bovine Serum, Qualified, Cat. 26140095), 2 mmol/L glutamine, and penicillin–streptomycin 100 U/mL, and kept in an incubator at 37°C in a humidified atmosphere containing 95% CO_2_. The culture medium was changed every 2 days.

Cells were subcultured once a week, by trypsinization, [Trypsin 0.25% (w/v)–0.53 mm EDTA] followed by gently repipetting, and plated at a density of 4 × 10^3^ cells per square centimeter. The culture medium was replaced every 3 days. For electrophysiological recording purposes, cells were plated on glass coverslips, previously placed in 35‐mm Petri dishes.

### Electrophysiological recording of cells

Membrane ion currents were recorded using the whole‐cell patch clamp technique following standard procedures, as described elsewhere (Ponce 2006). Briefly, micropipettes were produced by heating and pulling borosilicate glass tubing (cat. 34500‐99, Kimble Chase, USA) with a horizontal puller device (P‐87, Sutter Instrument Co. CA, USA). Tip resistance of micropipettes was from 2 to 5 MΩ after heat polishing. Micropipettes were backfilled with a saline solution (see Solutions) and attached through a pipette holder to a piezoelectric‐driven micromanipulator (PCS6000, Burleigh Co.). Mechanical manipulation of pipettes was monitored with an inverted microscope (Diaphot 300, Nikon, Japan). Glass coverslips containing HEK‐293 cells were immersed on a chamber containing an extracellular solution (see Solutions) and continuously perfused. A glass tubing filled with 2% agarose in 500 mmol/L KCl was set to made electrical contact between the bathing solution and the reference electrode, which was immersed in 500 mmol/L KCl. Voltage pulse protocols and recording of ion currents were made with a patch clamp amplifier (8900, DAGAN Corp, MN, USA) controlled by a dedicated software suite (pclamp 8.0, Axon Instruments Inc. CA, USA). Patch rupture was achieved by suction after gigaseal reached values greater than 2 GΩ (typically 5 GΩ). Unless otherwise stated, membrane potential protocol consisted of series of square pulses, which from a holding value of −120 mV, changed to a test potential from −60 mV to +80 mV in steps of 20 mV, then returning to a holding potential of −80 mV. A p/4 protocol was set to subtract linear components.

### Measurement of membrane capacitance

A capacitive current transient was induced by a hyperpolarizing square pulse of voltage, from −100 to −110 mV, and recorded at 10 KHz. Membrane capacitance was calculated offline by integrating the area of the capacitive transient at the onset of the pulse, then dividing the integrate by the amplitude of the pulse (−10 mV), according to the following equation:

$$c_{m}=\frac{\int_{t_{0}}^{\infty }I_{c}\cdot \Delta t}{\Delta V}$$

where *c*_m_ is the membrane capacitance, *I*_c_ the capacitive current, and Δ*V* the amplitude of the voltage pulse (−10 mV). Calculation of the integrate was made with the clampfit module of pClamp 8.0 (Molecular Devices).

### Solutions

Pipette (intracellular) solution was composed of (mmol/L): 135 K‐gluconate, 5 KCl, 1 MgCl_2_, 5 glucose, 10 HEPES, 10 EGTA, pH 7.4, adjusted with KOH. Extracellular solution composition consisted of (mmol/L): 140 Na‐gluconate, 5 K‐gluconate, 3 CaCl_2_, 1 MgCl_2_, 5 glucose, 10 HEPES, pH 7.4 adjusted with NaOH.

### Chemicals and drugs

All salts, chemicals, and drugs were purchased from Sigma‐Aldrich. Actinomycin D (A9415) was dissolved in DMSO (5 mg/mL) prior to use. Cycloheximide (C4859) was obtained as a ready‐made solution (100 mg/mL in DMSO).

### Statistical analysis

Descriptive statistics, significance tests, and ANOVA of single factor were made with the analysis module of EXCEL (Office 2003, Microsoft Co.). A minimal level of *α *= 0.05 was taken as statistically significant.

## Results

### Biophysical properties of endogenous potassium currents of HEK293 cells

**Figure 1:**
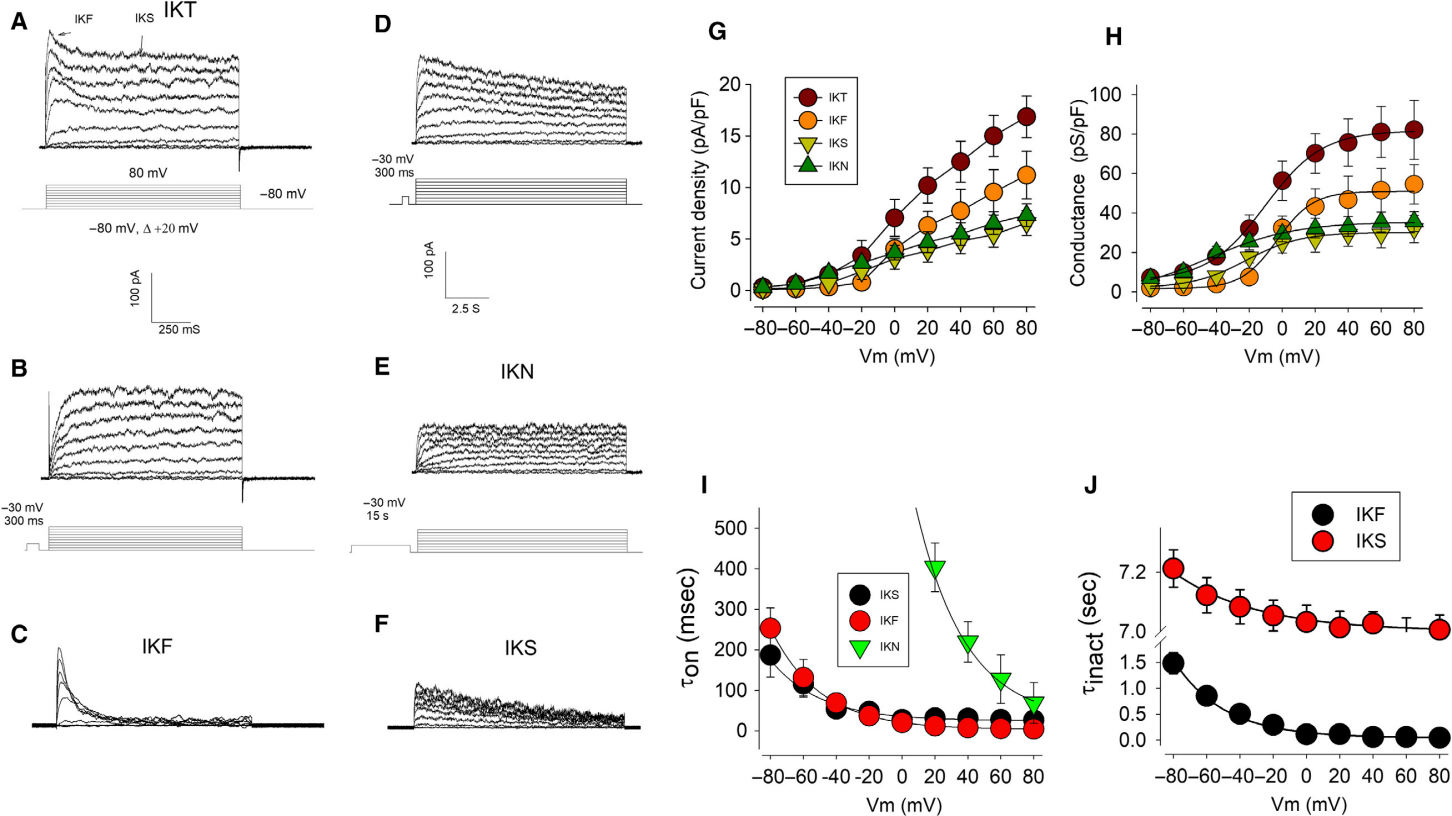
Endogenous ion currents from HEK293 cells. (A) Representative series of currents recorded from HEK293 cells in response to a voltage protocol as shown in the lower part. (B) Series of currents obtained with a protocol that includes a −30 mV prepulse. (C) Fast inactivating currents (IKF) are obtained by subtraction of traces as in B from traces as in A. (D) Currents recorded with the same protocol as in B but with the length of the pulse extended from 1 to 10 sec show slow inactivation. (E) A prepulse of −30 mV, lasting 1500 msec, reveals the noninactivating component. (F) The slowly inactivating currents are disclosed by subtracting current recordings as in D from those as in E. (G) Relationship between the current density and the test voltage is shown for IKT as well as for each of the functionally distinct components (IKF, IKS and IKN). (H) Relationship between the conductance and the test voltage for IKT and its components. (I) Voltage dependence of the activation kinetics for the distinct K current components. (J) Voltage dependence of the inactivation process for the fast and the slow inactivating components.

Ion currents were recorded from HEK cells in mature monolayers by the whole‐cell clamp technique. The protocol of stimulation consisted of a series of squared test pulses from −80 to +80 mV, lasting 1000 msec, in steps of 20 mV, from a holding of −120 mV. To exclude anion currents, chloride was substituted by gluconate on the external media composition (see Solutions). Figure 1A shows a representative series of traces of ion currents obtained under these conditions (IKT). Ion currents activate, increasing their magnitude over time until reaching a sustained level. Both the promptness of activation and the sustained magnitude increase with voltage. From about +20 mV, currents start displaying inactivation: Ion currents increase its magnitude over time until reaching a peak and then decline with a kinetics that can be fitted with two time constants, a fast and a slow (*as pointed out by arrows on Figure* 1 *A*). This multiple kinetics of the inactivating phase suggests that several functionally distinct components are contributing to shape such currents. This fact prompted us to discern such components with two alternate protocols of stimulation: First, a second series of test pulses was set, similar to the previously described; however, this time each test pulse was led by a prepulse of −30 mV during 500 msec, which was intended to inactivate the fast inactivating component (IKF). This component was further revealed by subtraction of currents, recorded with and without the prepulse (Fig. 1A–C). Second, to discern the slowly inactivating component (IKS), cells were first stimulated with a set of pulses similar to the previous one, but having the time length of each test pulse extended to 10 sec (Fig. 1D, bottom); immediately after, cells were stimulated with another series of pulses, of the same amplitude and length, but now each pulse was preceded by a pulse of −30 mV for 1.5 sec. A lapse of 10 sec was set between episodes to allow recovery of slow inactivation. This procedure excluded the inactivating components, leaving only noninactivating currents (IKN) as shown in Figure 1E. The slowly inactivating currents (IKS, Fig. 1F) were further revealed from the difference between traces, as in 1D, from traces as in 1E.

Figure 1G shows the relationship between the average current density (±S.E., pA/pF) and the test voltage from the total potassium current (IKT) as well as from each of its functional components. IKT (*cherry circles*) has an outward rectification profile that begins to be conspicuous at −60 mV, with a current density of 0.64 ± 0.5 pA/pF up to 16.8 ± 2.0 pA/pF at +80 mV. IKF(*orange circles*), the fast inactivating component, starts to be noticeable until 0 mV with a magnitude of 4.05 ± 1.1 pA/pF that increases to 11.2 ± 2.3 pA/pF at +80 mV. IKS (*light green triangles*), the slowly inactivating component, starts to be manifest at −20 mV with 1.85 ± 0.8 pA/pF rising to 6.5 ± 1.2 pA/pF at +80 mV. Finally, IKN (*dark green triangles*), the noninactivating component, starts to be noticeable from around −40 mV with 1.7 ± 0.5 pA/pF up to 7.3 ± 1.1 pA/pF at +80 mV. Figure 1H shows the relationship between the mean conductance density (pS/pF) and the test potential of all distinct components of current. These data were fitted with the Boltzmann equation:

$$g=g_{0}+\frac{g_{\max}}{1+{\text{e}}^{-\left(\frac{v-v_{1/2}}{k}\right)}}$$

where *g*_max_ is the maximal conductance, *v*_1/2_ is the test potential producing half conductance activation, and *k* is a parameter determining the stepness of voltage dependence. IKT was fitted with *g*_max_ of 75 ± 2 pS/pF and a *v*_1/2_ of −10.3 ± 1.3 mV; IKF was fitted with *g*_max_ of 49 ± 2.9 pS/pF and a *v*_1/2_ of −3.1 ± 2.2 mV, b 10.8; For IKS, *g*_max_ was 28 ± 1.9 pS/pF and *v*_1/2_ = −21.8 ± 3.3 mV. IKN was fitted with *g*_max_ of 37 ± 6.6 pS/pF and *v*_1/2_ of −45.2 ± 9.5 mV.

For each current component, the time constant of activation (*τ*_on_) was obtained by fitting the rising phase of current traces at each test voltage. Figure 1I shows that for all the three components of current (IKF, IKS, and IKN), the relationship between *τ*_on_ and the test voltage follows an exponentially decaying trend. For IKF, *τ*_on_ ranges from 253 ± 50 msec at −80 mV to 4 ± 2 msec at +80 mV; for IKS, 186 ± 50 msec to 26 ± 5 msec; and for IKN 10032 ± 120 msec to 68 ± 50 msec. The time constant of inactivation (*τ*_inact._) was obtained by fitting the decaying phase of current traces (Fig. 1J). It ranged from 1480 ± 220 to 46 ± 10 msec for IKF, whereas for IKS it was from 7212 ± 220 to 7000 ± 90 msec, which resulted to be nonsignificantly distinct (*P* > 0.05, ANOVA one‐way).

**Figure 2:**
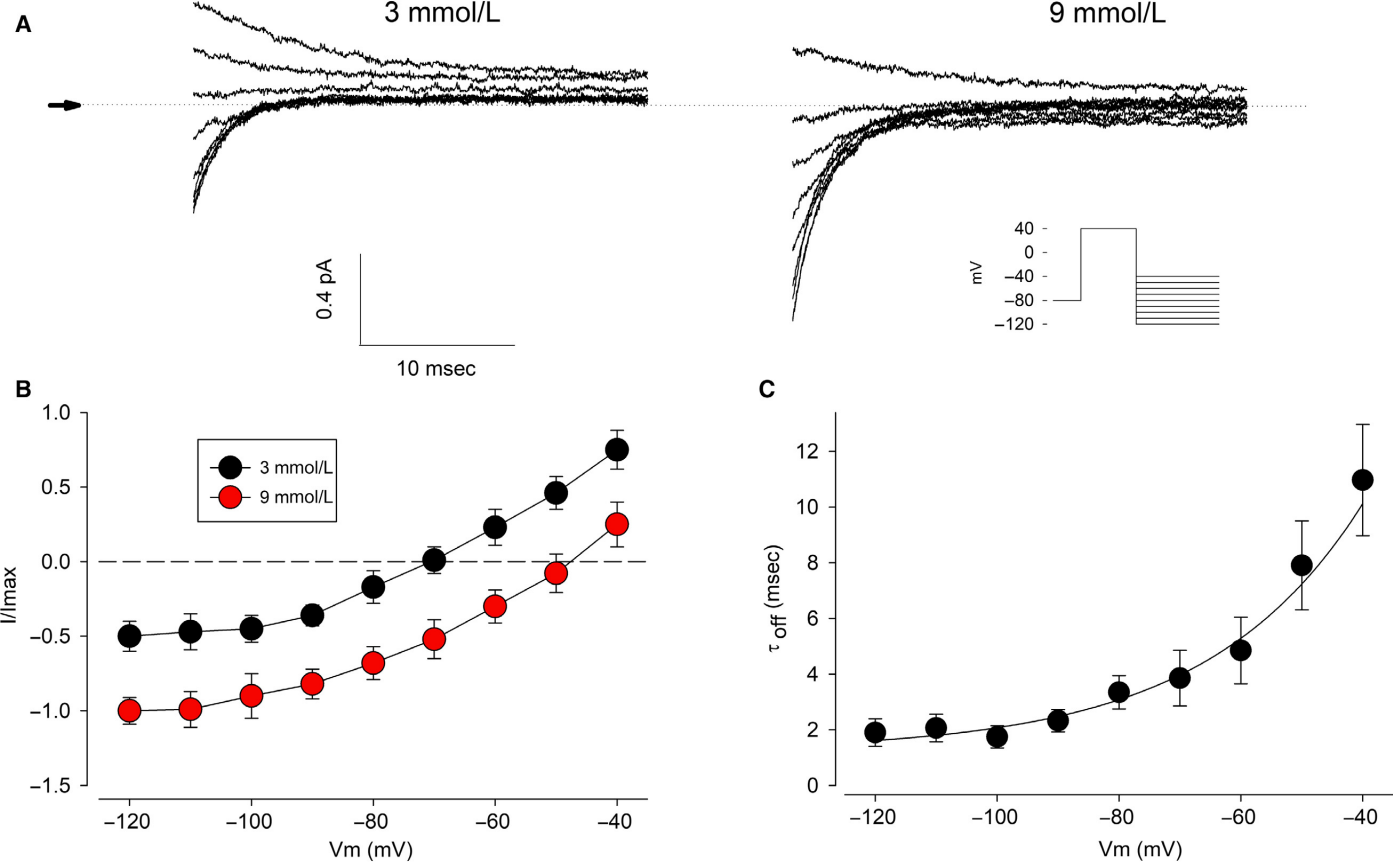
Potassium selectivity of endogenous ion currents from HEK293. (A) representative examples of tail current series, induced by the voltage protocol shown on the inset, to estimate the reversal potential under distinct external potassium concentrations. (B) The I–V relationship is shifted by changes in the external potassium concentration, as expected for a K selective conductance. (C) Voltage dependence of the time constant of deactivation.

In order to verify the selectivity of these currents, we designed a protocol, aimed to induce tail currents: starting from a holding of −80 mV, voltage was switched to +40 mV for 1 sec, to activate IKT currents, then to a varying test voltage from −120 to −40 mV in steps of 10 mV. From the relationship between the initial magnitude of tail currents and the test voltage, the reversal potential (Er) was interpolated. As Figure 2 (A and B) shows, Er shifts from −70 mV when external potassium concentration is 3 mmol/L to −48 mV when potassium is raised to 9 mmol/L. These results, therefore, indicate that such currents are selective to potassium. Figure 2C shows the relationship between the time constant of deactivation and the test voltage. It follows a simple, exponential trend, and ranges from 1.9 ± 0.5 msec at −120 mV to 11 ± 2 msec at −40 mV.

### Pharmacological properties of endogenous K currents of HEK‐293 cells

**Figure 3:**
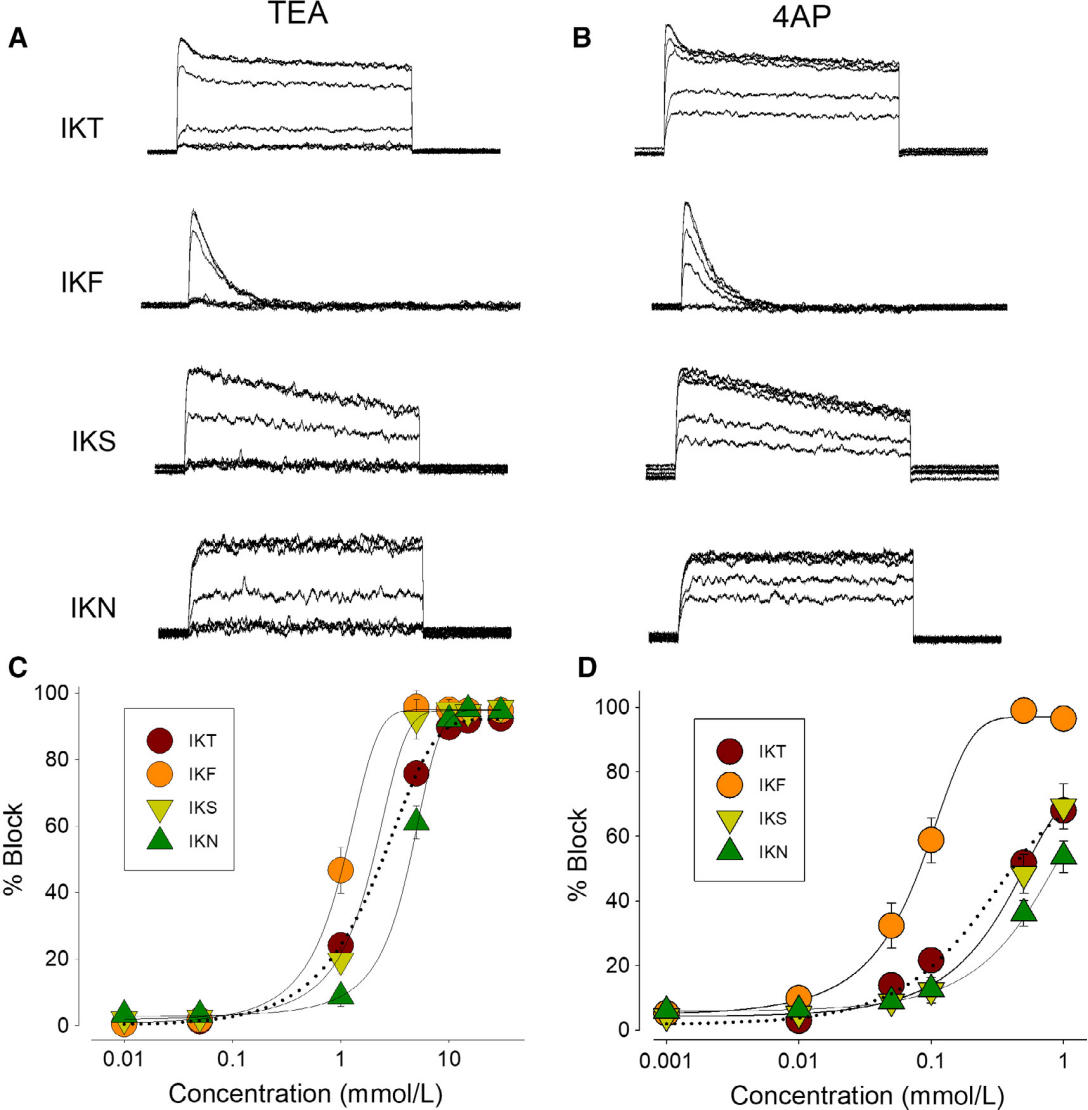
Pharmacological properties of endogenous K currents of HEK‐293 cells. Effect of TEA and 4AP. (A and B) Representative recordings showing the effect of TEA (*left column*) and 4AP (*right column*) on endogenous potassium currents IKT, and its components (IKF, IKS, and IKN). All currents were obtained by test pulses of +60 mV. (C and D) Semilog plots showing the dose–effect relationship.

We analyzed the effect of TEA and 4AP, which are general K channel blockers, on IKT as well as on each one of its components. For this purpose, we recorded current traces, induced by test pulses of +60 mV, from cells incubated with progressively increasing concentrations of blockers in the external media. As Figure 3 shows, both compounds effectively reduced the size of total K currents as well as its components, although with distinct sensitivity. To quantify such sensitivity, a percent blocking index (%*B*_d_) was calculated for each drug concentration with the following transformation:

$$\%B_{d}=\frac{I_{0}-I_{d}}{I_{0}}\times 100$$

Where *I*_d_ is the peak magnitude of ion currents at a given drug concentration, whereas *I*_0_ is the corresponding value with no blocker added. Plotting of these values versus the log_10_ of blocker concentration follows a sigmoidal relationship that was fitted with the following function:

$$\%B_{x}=\frac{B_{\max}}{1+e^{-\left(\frac{x-x_{50}}{b}\right)}}$$

where *B*_max_ is the maximal blocking effect of the drug, *x* is a given testing drug concentration, and *x*_50_ the concentration blocking half the amplitude of currents.

TEA produced a *B*_max_ of 91% on IKT with an *x*_50_ of 2.9 ± 0.5 mmol/L (*n* = 10); On IKF, *B*_max_ was 95% with *x*_50_ of 1.0 ± 0.005 mmol/L (*n* = 9); a similar result was found for IKS, with *B*_max_ of 94% and *x*_50_ of 1.5 ± 0.1 mmol/L (*n* = 9). IKN was blocked with *B*_max_ of 94% and *x*_50_ of 4.19 ± 0.05 mmol/L (*n* = 10). 4AP also blocked IKT currents, although it produced a lower maximal effect (68%) than TEA, a lower concentration was needed to produce a half effect(*x*_50_ = 0.3 ± 0.08 mmol/L, *n* = 10); It blocked more efficiently IKF (*B*_max_ = 97.5%, *x*_50_ = 0.08 ± 0.005 mmol/L, *n* = 9) than IKS (*B*_max_ = 70%, *x*_50_ = 0.37 ± 0.01 mmol/L, *n* = 9) and IKN (*B*_max_ = 55.8%, *x*_50_ = 0.37 ± 0.01 mmol/L, *n* = 10).

**Figure 4:**
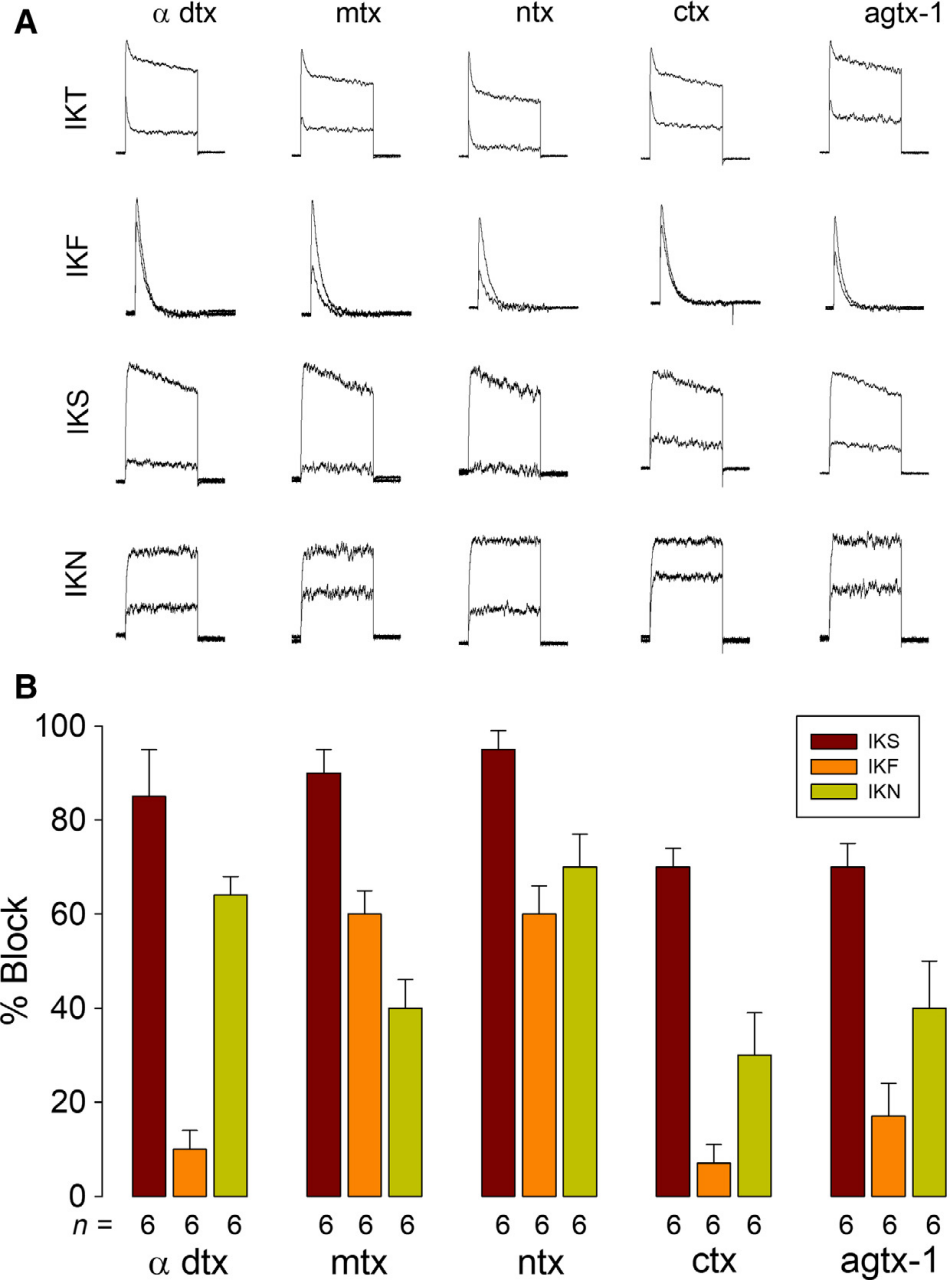
Pharmacological properties of endogenous K currents of HEK‐293 cells. Effect of Kv1 blockers. (A) Representative recordings of currents at +60 mV (IKT,IKF,IKS, and IKN) before and after addition of toxins. (B) Statistical analysis showing the percentage blocking effect of each toxin.

In addition to TEA and 4‐AP, we examined the effect of a set of toxins that has been described as specific blockers of molecular entities of voltage‐dependent potassium channels of the Kv1 subfamily: *α*‐dendrotoxin targets Kv1.1, Kv1.2, and Kv1.6 (Harvey 2001); noxiustoxin, a potent blocker of Kv1.2 and Kv1.3; charybdotoxin, a potent blocker of K_Ca_1.1, Kv1.2, and Kv1.3 (Grissmer et al.^1994^); agitoxin‐1, which targets Kv1.3 (Garcia et al. 1994); and margatoxin, a specific blocker of KV1.3 and KV1.6 (Leonard et al. 1992; Garcia‐Calvo et al. 1993). We added those toxins (a single concentration) to the external solution and compared the magnitude of the peak current at +60 mV before and after its addition. Figure 4A shows a representative example of the effect of these toxins on IKT as well as on its functional components. Figure 4B shows the averaged % blocking effect that these toxins produce on each functional component. *α*‐dendrotoxin (50 nmol/L) blocked IKF (10 ± 10%), IKS (80 ± 4%), and IKN (45 ± 4%); margatoxin (0.5 nmol/L) blocked IKF (15 ± 5%), IKS (70 ± 5%), and IKN (40 ± 6%); noxioustoxin (100 nmol/L) blocked IKF (25 ± 4%), IKS (65 ± 6%), and IKN (50 ± 7%); charybdotoxin (15 nmol/L) blocked IKF (21 ± 4%), IKS (85 ± 4%), and IKN (30 ± 9%). Finally, agitoxin‐1 (50 nmol/L) blocked IKF (35 ± 5%), IKS (75 ± 7%), and IKN (40 ± 10%).

### Influence of culture conditions on endogenous K currents

Next, we evaluated whether culture conditions affect the expression of endogenous K currents. For this purpose, we analyzed how these currents are modified by changes in the passage number, the cell density, the substrate, and the serum complementing the culture media. In order to compare the magnitude of currents at distinct values of each condition, we recorded IKT as well as IKF, IKS, and IKN currents in response to a test pulse of +60 mV from a number of HEK293 cells to make statistical analysis.

#### Passage number

**Figure 5:**
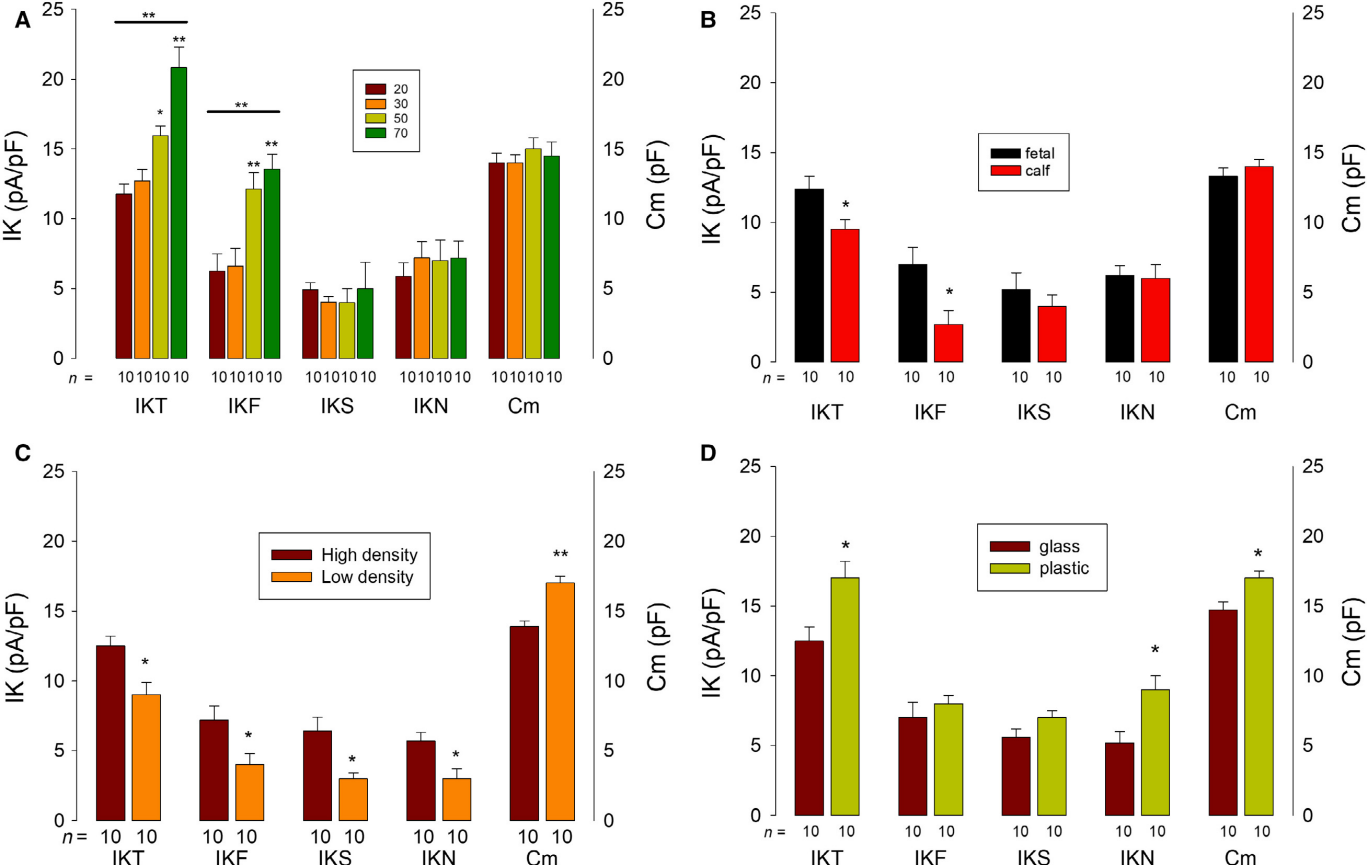
Influence of culture factors on endogenous K currents of HEK‐293 cells. (A) Statistical analysis, comparison of the magnitude of endogenous K currents at +60 mV from cells at passage numbers 20, 30, 50, and 70. The line above groups of bars IKT and IKF denotes a significant difference among bars of the same group (*P* < 0.001, ANOVA). (B) Comparison of the magnitude of endogenous K currents at +60 mV of cells incubated with fetal serum and cells incubated with calf serum. (C) Comparison of the magnitude of endogenous K currents at +60 mV of cells plated at high versus low density. (D) Comparison of the magnitude of endogenous K currents at +60 mV of cells plated on glass coverslips and of cells plated on the surface of the Petri dish. A (*) above bars denotes a statistically significant difference of (*P* < 0.05).

We compared K currents from cells at passage numbers 20, 30, 50, and 70. As shown on Figure 5A, we found that IKT increase significantly (*P* < 0.001, ANOVA) from 11.8 ± 1.7 pA/pF at passage 20 to 20.8 ± 1.4 pA/pF at passage 70. This increase seems to be mainly due to IKF, whose magnitude increased significantly (*P* < 0.005, ANOVA) from 6.26 ± 1.23 pA/pF at passage 20 to 13.5 ± 1.1 pA/pF at passage 70. The slow inactivating and the noninactivating currents did not change significantly.

#### Serum containing media

We compared K currents from cells grown on culture media complemented with fetal bovine serum (Gibco™ Fetal Bovine Serum, Qualified, Cat. 26140095) versus that from cells grown on media complemented with calf serum (HyClone™Newborn Calf Serum, GE Healthcare Life Sciences, cat. # SH30118.03). As Figure 5B shows, the IKT average current density of cells cultured with calf serum (9.6 ± 0.7 pA/pF) was significantly smaller (*P* < 0.05) than that of cells cultured with fetal bovine serum (12.4 ± 0.9 pA/pF). IKF was also significantly lower (*P* < 0.01) in cells grown with calf serum (2.7 ± 1.1 pA/pF) than in cells grown with fetal serum 7.0 ± 1.2 pA/pF, whereas IKS and IKN were not significantly different.

#### Cell density

We compared cells seeded at low (5 × 10^2^ cells/cm^2^) versus high density (3 × 10^4^ cells/cm^2^). Both batches of cells were harvested from the same flask, counted, and plated separately. Ion currents were recorded 24 h after seeding (Fig. 5C). We found that the membrane surface of cells seeded at high density was significantly (*P* < 0.01) lower (14.0 ± 0.3 pF) than that of cells seeded at low density (17 ± 0.5 pF). Likewise, the average IKT current density was significantly smaller from cells plated at low density than from cells plated at high density (*P* < 0.05). The mean current density of all three functional components (IKF, IKS, and IKN) was significantly smaller from cells seeded at high density than from cells seeded at low density.

#### Substrate

Endogenous K currents, as well as membrane surface, of cells plated on plastic were compared with that of cells plated on glass. For this purpose, cells were seeded on 60‐mm Petri dishes containing glass coverslips at 4 × 10^3^ cells/cm^2^. Recordings were made 24 h after plating, either on cells deposited on glass coverslips or on the surface of the Petri dish. As Figure 5D shows, the membrane surface of cells seeded in plastic was significantly (*P* < 0.05) larger than in cells seeded in glass. Likewise, the IKT current density of cells seeded on a plastic was significantly higher (*P* < 0.05) than that of cells plated on glass. A significant increase (*P* < 0.05) was observed in the noninactivating component, whereas no significant difference was observed, neither in IKF nor in IKS.

### Influence of cell–cell contact on the expression of HEK endogenous voltage‐gated potassium channels

**Figure 6:**
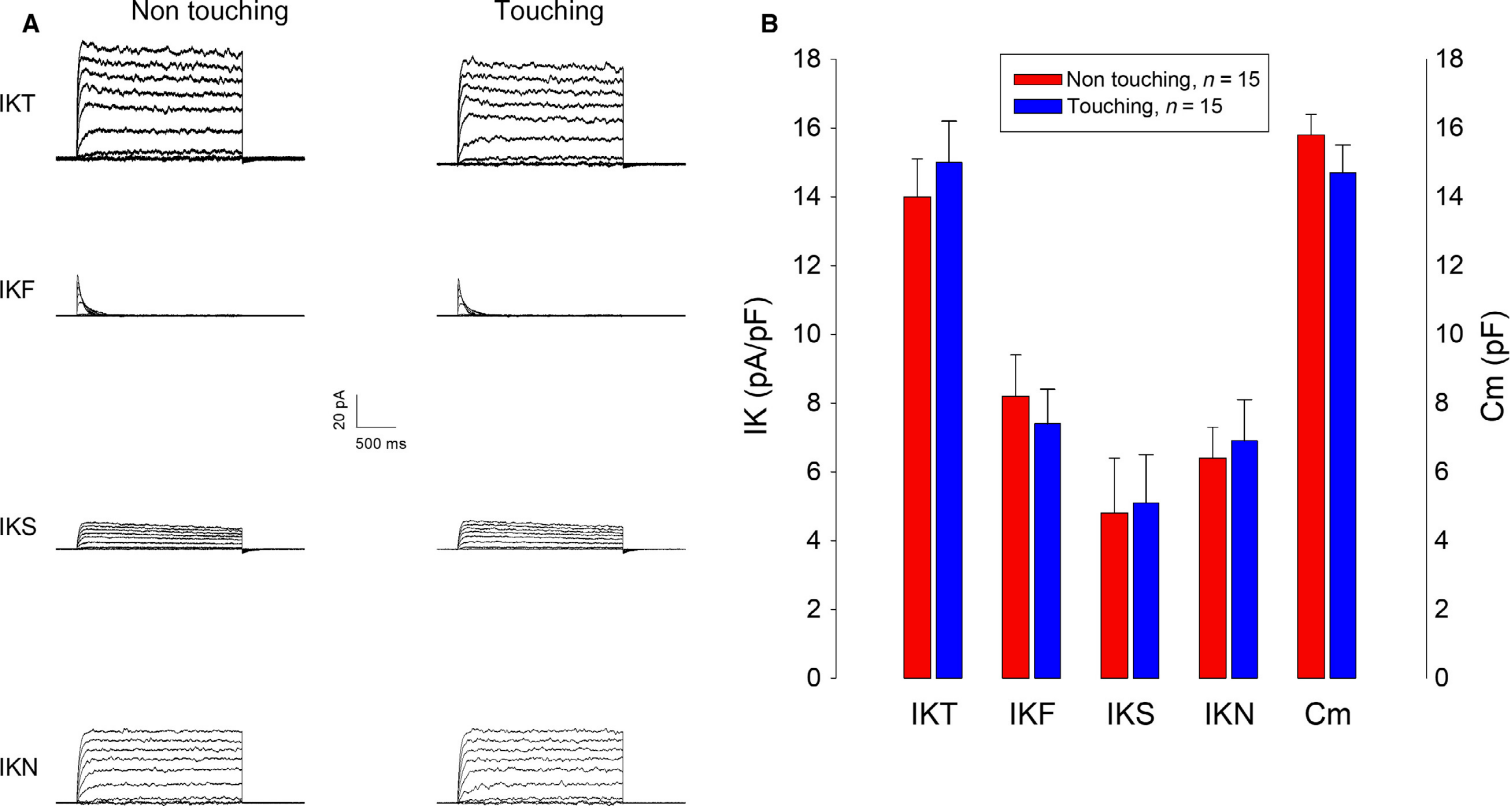
Influence of cell–cell contact on endogenous K currents of HEK‐293 cells. (A) Representative series of IKT, IKF, IKS, and IKN currents from touching and nontouching HEK cells. (B) Comparison of the mean values of each potassium current component (IKT, IKF, IKS, and IKN) and membrane capacitance shows no statistically significant difference.

To determine if cell–cell contact influences the expression of voltage‐dependent potassium channels, we made whole‐cell recordings of cells that were either isolated or in contact with neighboring cells and compared the magnitude of IKT currents, as well as its components (IKF, IKS, and IKN). Recordings were made 6 h after plating. Touching and no touching cells were recorded alternatively from a same coverslip containing seeded cells. Figure 6A shows representative series of currents of each condition. As Figure 6B shows, we found no significant difference, neither in the mean value of the membrance surface nor in IKT or any of its components. These results suggest therefore that cell–cell contact does not influence the expression of HEK293 endogenous voltage‐gated potassium currents.

### Effect of trypsinization on endogenous K currents

**Figure 7:**
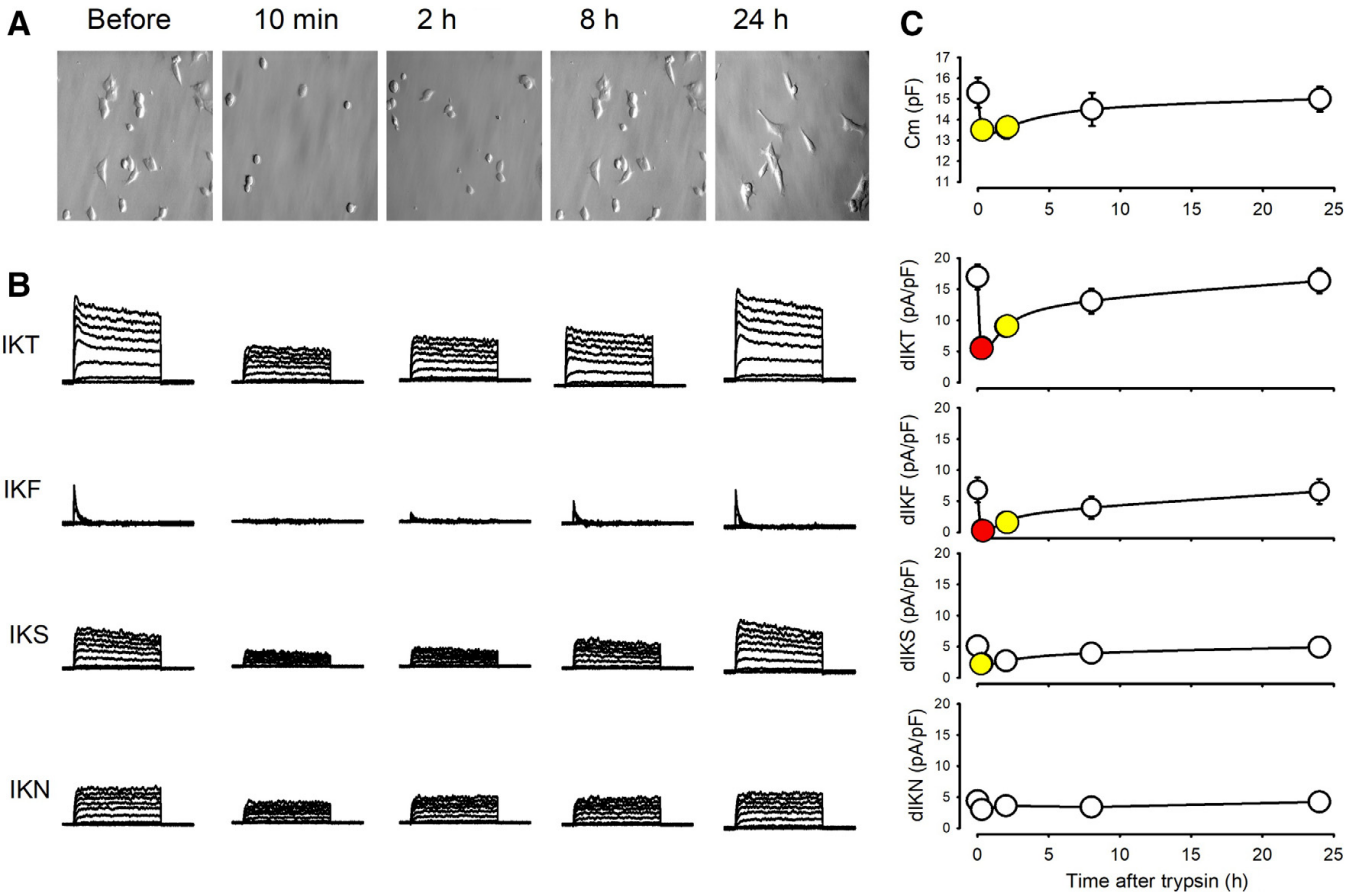
Effect of trypsin on endogenous K currents of HEK‐293 cells. (A) Set of five columns showing (from top to bottom) a representative image of cells in culture at distinct times, before and after treatment with trypsin. (B) Representative series of currents (IKT, IKF, IKS, and IKN) at distinct times after trypsin treatment. (C) Plots showing how trypsin treatment decrease the average value of membrane capacitance (top) and the current density of endogenous K currents and its components, and how they recover over time. Red and yellow dots indicate a statistically significant difference of (*P* < 0.005) and (*P* < 0.05), respectively.

In order to subculture, HEK293 cells, as most cultured cells, are typically incubated with trypsin–EDTA followed by gentle mechanical dissociation. Because of this procedure, cells detach from its substrate, acquiring a spherical shape, but after seeding, they reattach to the substrate and resume its shape. To evaluate how this treatment affects K currents, we recorded ion currents from cells, as early as 10 min after trypsinization and at subsequent times up to 24 h. Figure 7A shows representative series of each current component, whereas Figure 7B shows the time course of the membrane capacity and the density of current of each component (at +60 mV) before (at time 0) and up to 24 h after trypsinization. After treatment, the membrane capacitance of cells recently plated is significantly reduced (*P* < 0.05). IKT currents also change its magnitude and shape, as they lose the transient peak that is typically recorded from cells before tripsinization. Nonetheless, both features are recovered progressively over time and, after about 24 h of trypsinization, IKT currents recover its original shape and size.

In addition to IKT, we analyzed how trypsinization affects each one of the functional components of K currents. IKF decreases significantly (*P* < 0.001) from 6.8 ± 2.0 (*n* = 14) before to 0.5 ± 0.1 (*n* = 12) pA/PF at 10 min after trypsinization; a significant reduction is also observed on IKS (*P* < 0.05), albeit not as pronounced as IKF, from 5.1 ± 1.2 (*n* = 13) to 2.3 ± 0.8 (*n* = 10) pa/PF. IKN did not change significantly from 4.4 ± 1.2 (*n* = 14) to 3.0 ± 0.8 (*n* = 12) pA/pF. It is clear from these observations that the subculturing procedure reduces the size and changes the profile of endogenous K currents, affecting most notoriously IKF, the fast inactivating component and to a less degree IKS, the slow inactivating one.

**Figure 8:**
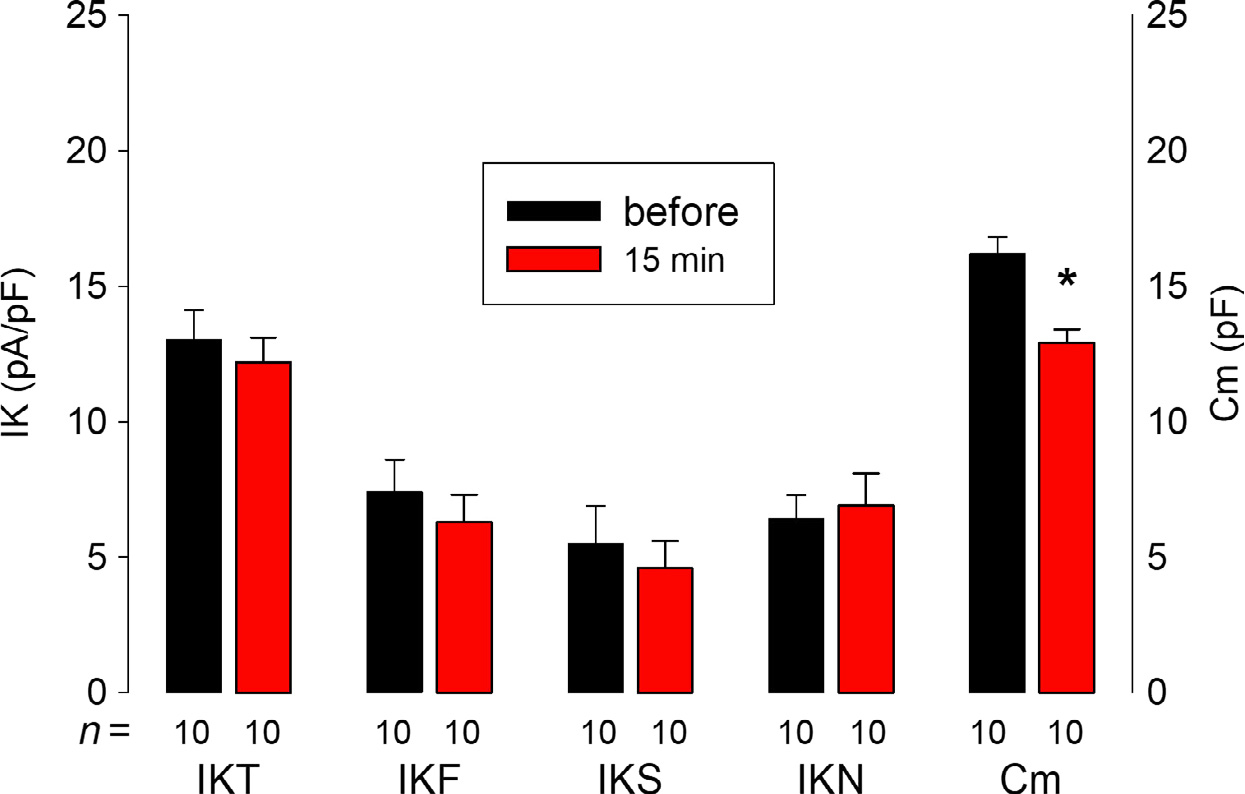
Mechanical detachment of cells does not reduce current density. Graph shows the average value of membrane capacitance (Cm) and endogenous currents before and 15 min after mechanical detachment of cells, without trypsin. As shown, this treatment did not change the current density but the membrane capacitance.

This reduction could be simply due to retrieval of the plasma membrane during the harvesting process but, if that were the case, the density of the channels would be expected to remain unchanged. To probe this possibility, we discarded trypsin out of the harvesting process, detaching cells only by gentle repippeting, and assayed how the membrane surface and endogenous K currents are affected. As Figure 8 shows, although membrane surface was significantly reduced by mechanical harvesting, no significant reduction was observed, neither in the average density of IKT nor in any of its components. Therefore, our results lead us to think that trypsin is selectively degrading some types of potassium channels from those that produce endogenous currents.

### Effect of actinomycin D and cycloheximide on the recovery of surface membrane and potassium currents after tripsinization

**Figure 9:**
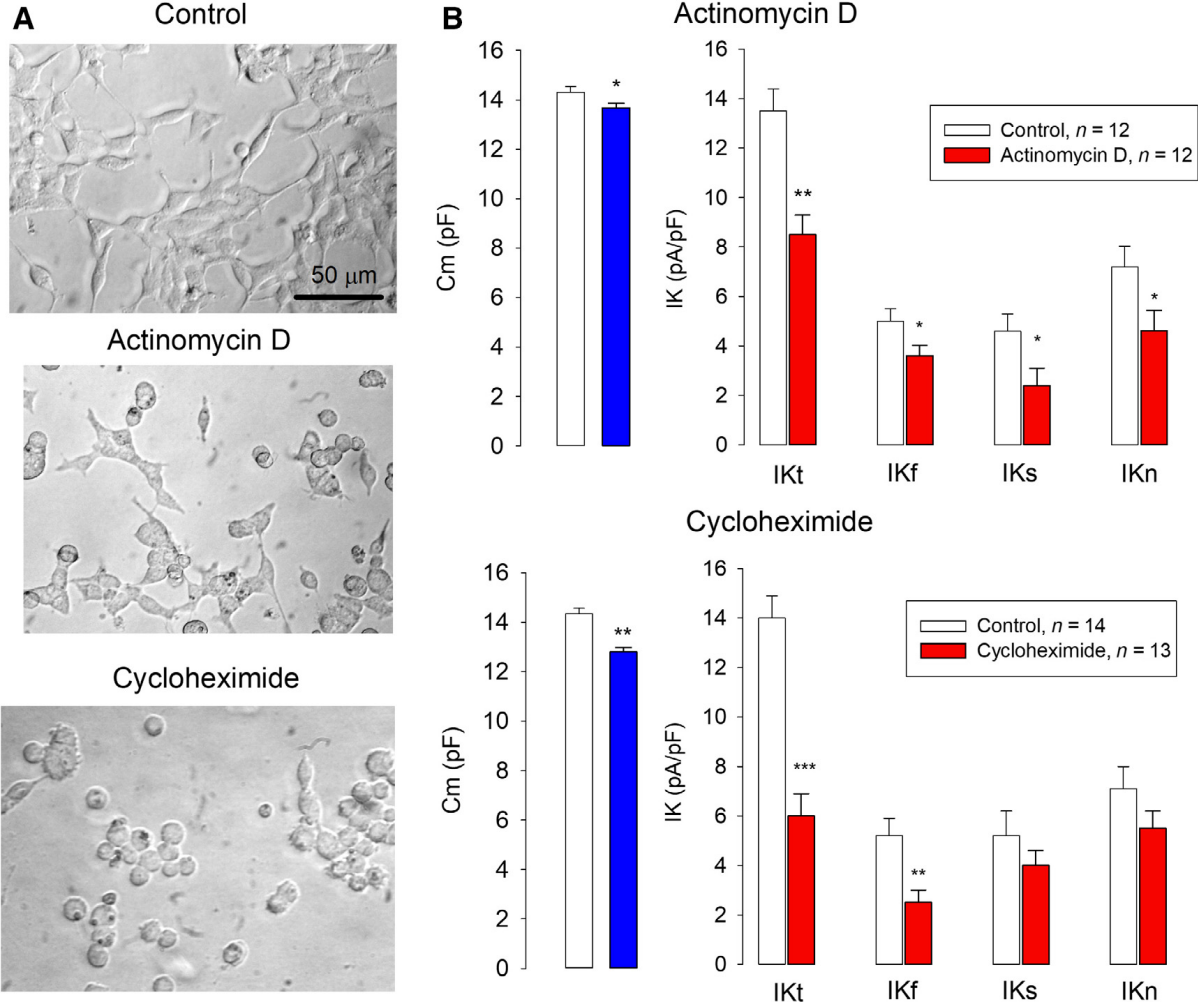
Recovery of K currents after trypsinization requires synthesis of new channels. (A) Representative images of cultured HEK cells, obtained 5 h after trypsinization and seeding. (B) Bars comparing the membrane capacitance (left) and current density of IKT, IKF, IKS, and IKN under control conditions versus treatment with actinomycin D (upper) and cycloheximide (lower). *, **, and *** denotes a statistically significant difference (*P* < 0.05, 0.01, and 0.005), respectively.

To find out whether new RNA and/or protein is required for recovering of K currents after trypsinization, we studied the effect of actinomycin D (5 *μ*g/mL) and cycloheximide (10 *μ*g/mL), drugs that inhibit the synthesis of RNA (Graham et al. 1977; Liantonio et al. 2016) and protein (Sobell, 1985; Schneider‐Poetsch et al., 2010), respectively. For this purpose, cells in mature monolayers were trypsinized and inhibitors were added to the culture medium immediately after plating. The membrane capacitance and density of current of total (IKT) and each component (IKF, IKS, and IKN), from both control and treated cells, were measured, 5 hours after trypsinization. Cells treated with actinomycin D recovered partially its shape and adherence (Fig. 9A, *middle*), yet its membrane surface was significantly lower (*P* < 0.01) than control cells (Fig. 9B, *upper left*). This treatment impaired significantly (*P* < 0.01) the recovery of total endogenous K currents, as well as each of its components: IKF, IKS, and IKN. (Fig. 8B, *upper right*). Cycloheximide produced a more dramatic effect than actinomycin D: most cells remained spherical and barely attached to the substratum (Fig. 8A, *bottom*), this was reflected in a significantly lower membrane surface, as compared to control cells (Fig. 8B, *bottom left*). The recovery of the density of IKT currents was more severely impaired (*P* < 0.001) by cycloheximide than by actinomycin D (Fig. 8B, *bottom right*), mostly due to the fast (IKF) component, whose recovery was significantly impaired (*P* < 0.01), whereas the recovery of the other components was not significantly affected.

## Discussion

HEK293 is a cell line of striking interest because is used as a model to investigate the functional properties of exogenous proteins, including ion channels. In this work, we describe the biophysical and pharmacological properties of HEK′s endogenous, voltage‐dependent potassium currents, and show that its magnitude and shape depend on culture conditions, and that the treatment of cells with trypsin, notoriously reduces those currents. It is worth noticing that HEK cells express A‐type K currents, which are commonly expressed in excitable cells, such as neurons and cardiomyocytes. Although HEK293 are regarded as epithelial, given its origin from human embryonic kidney (Graham et al. 1977), new evidence has emerged suggesting that these cells have instead a neuroendocrine origin derived from the adrenal gland (Shaw et al., 2002; Vetter and Lewis 2010). Our finding that HEK cells express A‐type K currents reinforces such suggestion. We show in this work that the magnitude and shape of endogenous potassium currents are related to culture factors such as the passage number, the media composition, and the substrate on which cells are seeded. This in part may account for the discrepancy in the shape of endogenous currents that has been reported previously (Yu and Kerchner 1998; Zhu et al. 1998; Avila et al. 2004; Varghese et al. 2006; Kurejová et al. 2007). We found that A‐type currents was the most sensitive component, depending on the passage number, as well as the media containing serum and cell density at plating, whereas the delayed rectifier‐type currents were less sensitive to such variations, depending only on the cell density and the substrate.

Our results indicate that both 4AP and TEA block endogenous voltage‐dependent K currents although with distinct sensitivity. Both compounds are nonspecific K channel blockers, yet 4AP blocks more efficiently IA that delayed rectifying‐type currents. On the other hand, the results obtained with the toxins indicate that subunits of the Kv1 subfamily contribute importantly to the shape and magnitude of IKT. This is in agreement with a previous work, which describes that the transient outward KV channels (IA) are due to expression of [KV1.4, KV3.3, KV3.4, and KV4.1] as well as a KV*β*2 subunit, whereas the delayed rectifier currents are produced by voltage‐dependent subunits [KV1.1, KV1.2, KV1.3, KV1.6, and KV3.1] (Jiang et al. 2002). We also showed that after incubation with trypsin–EDTA, HEK cells reduce its membrane surface as well as the density of K currents, more notoriously the fast inactivating component. The fact that a significant reduction was not observed when cells were detached by simple mechanical enforcement indicates that trypsin degrades an important amount of membrane K channels. We found also that cells restitute both plasma membrane and K currents within a few hours and, the fact that such restitution is abolished by actinomycin D and cycloheximide, suggest that they have to synthesize new channels to restore its former conditions. This process is interesting, because it provides a way to reduce the amount of endogenous K channels, hence minimizing the chance of unexpected results when HEK293 are used for heterologous expression assays. In addition, the fact that HEK cells restitute such channels after trypsinization indicates that HEK are able to regulate the density and variety of its endogenous channels; therefore, they could be a model to study the cellular and molecular mechanisms involved in the regulation of the expression of membrane channels.

In summary, HEK293 cells have been taken as a host for testing the expression of heterologous proteins, whereas its endogenous components had been generally neglected, however, as is clear from this study, endogenous voltage‐dependent K currents are not to be neglected, as they could interfere with heterologous K channels.

## Conflict of Interest

None declared.
